# Micronutrient Requirements for Growth and Hydrocarbon Production in the Oil Producing Green Alga *Botryococcus braunii* (Chlorophyta)

**DOI:** 10.1371/journal.pone.0041459

**Published:** 2012-07-25

**Authors:** Liang Song, Jian G. Qin, Shengqi Su, Jianhe Xu, Stephen Clarke, Yichu Shan

**Affiliations:** 1 School of Biological Sciences, Flinders University, Adelaide, Australia; 2 School of Animal Science and Technology, Southwest University, Chongqing, P. R. China; 3 Key Laboratory of Marine Biotechnology of Jiangsu Province, Huaihai Institute of Technology, Lianyungang, P. R. China; 4 School of Chemistry, Physics and Earth Sciences, Flinders University, Adelaide, Australia; 5 CAS Key Laboratory of Separation Science for Analytical Chemistry, National Chromatographic Research and Analysis Centre, Dalian Institute of Chemical Physics, Chinese Academy of Sciences, Dalian, P. R. China; US Dept. of Agriculture – Agricultural Research Service (USDA-ARS), United States of America

## Abstract

The requirements of micronutrients for biomass and hydrocarbon production in *Botryococcus braunii* UTEX 572 were studied using response surface methodology. The concentrations of four micronutrients (iron, manganese, molybdenum, and nickel) were manipulated to achieve the best performance of *B. braunii* in laboratory conditions. The responses of algal biomass and hydrocarbon to the concentration variations of the four micronutrients were estimated by a second order quadratic regression model. Genetic algorithm calculations showed that the optimal level of micronutrients for algal biomass were 0.266 µM iron, 0.707 µM manganese, 0.624 µM molybdenum and 3.38 µM nickel. The maximum hydrocarbon content could be achieved when the culture media contained 10.43 µM iron, 6.53 µM manganese, 0.012 µM molybdenum and 1.73 µM nickel. The validation through an independent test in a photobioreactor suggests that the modified media with optimised concentrations of trace elements can increase algal biomass by 34.5% and hydrocarbon by 27.4%. This study indicates that micronutrients play significant roles in regulating algal growth and hydrocarbon production, and the response surface methodology can be used to optimise the composition of culture medium in algal culture.

## Introduction

Microalgae have recently been receiving much attention in an attempt to explore their use as a potential feedstock for biofuel production [1], [2]. *Botryococcus braunii* is a green colonial microalga found in freshwater lakes, reservoirs, and ponds [3], [4] and is classified into A, B and L races depending on the type of hydrocarbons synthesized [5]. Race A produces C_23_–C_33_ odd numbered *n*-alkadienes, mono-, tri-, tetra-, and pentaenes and race B produces C30–C37 triperpenes while race L produces C40 tetraperpenes [5]. This species is characterised by a conspicuous ability to synthesise and accumulate a variety of hydrocarbons [6], [7], [8]. These hexane-soluble hydrocarbons have the potential to be converted into biofuels by catalytic cracking [9]. However, the great variation of hydrocarbon content in *B. braunii* (0.1∼86% of dry weight) provides an opportunity to explore the optimal growing conditions to maximise hydrocarbon production for a given *B. braunii* strain [10], [11], [12]. Therefore, it is necessary to identify the most efficient growing conditions for sustainable mass and hydrocarbon production in *B. braunii*.

The requirements for macronutrients by *B. braunii* have been intensively studied in the past a few decades. Largeau *et al*. [13] pointed out that the phosphorus (0.46 mM) in the Chu 13 medium was not limiting through the stationary growth phase in *B. braunii*, while the nitrogen concentration of 0.5 mM NO_3_^-^ is only adequate to sustain the growth of *B. braunii* for 10 days and the initial concentration of 8 mM NO_3_^-^ is required to maintain the growth of growth *B. braunii* for 35 days. Ammonia can inhibit botryococcene biosynthesis in the *B. braunii* race B [14], but the replacement of nitrite nitrogen for nitrate nitrogen benefits the growth of race A *B. braunii* [15]. Air enriched with 1% CO_2_ can enhance algal growth by doubling algal biomass and achieving 5-fold hydrocarbon production compared to aeration without CO_2_ enrichment [16]. Dayanada *et al*. [17] reported that the N: P ratio played a significant role in both biomass and hydrocarbon production in *B. braunii* and the N: P ratio of 1∶4 by weight favoured hydrocarbon production while the N:P ratio of 1∶0.5 by weight increased the yield of algal biomass.

**Figure 1:**
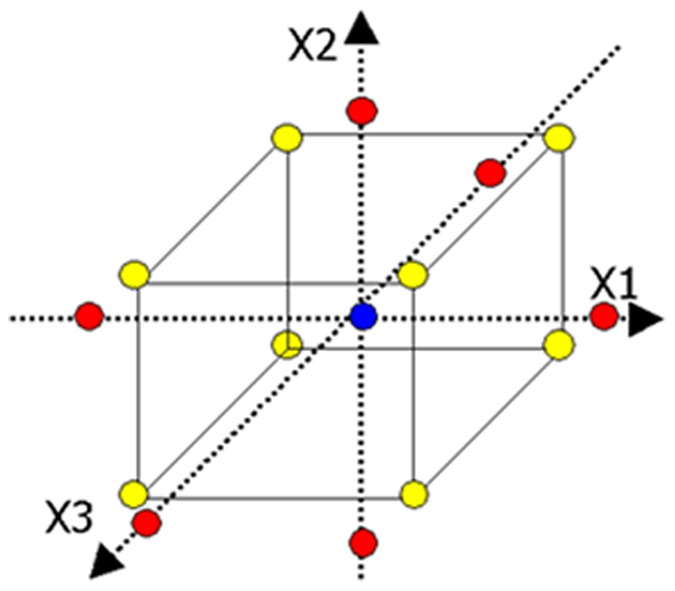
Illustration of the central composite design (only 3 out of the 4 dimensions are shown).

**Table 1:** Coded and actual values of experimental variables used in the central composite experimental design.

Independentvariables	Symbols			Levels		
		−1.72*	−1	0	1	1.72*
Fe (µM)	*x_1_*	0.03	2.39	5.35	8.31	10.44
Mn (µM)	*x_2_*	0.02	2.67	6.36	10.05	12.70
Mo (µM)	*x_3_*	0	0.13	0.31	0.50	0.62
Ni (µM)	*x_4_*	0	0.71	1.69	2.68	3.39

* Alpha values used for the axial points in this study.

Given the depth of understanding in the growth requirement for macronutrients in *B. braunii*, it is surprising that the requirements for trace elements are little known. Trace elements such as iron, molybdenum and manganese can play critical roles in a variety of metabolic pathways involving utilization of light, nitrogen, phosphorus, and CO_2_ [18], [19]. Among trace elements, iron is essential for photosynthetic electron transport, respiratory electron transport, nitrate and nitrite reduction, and detoxification of reactive oxygen species [20], [21], [22]. Mojaat *et al*. [23] demonstrated that the addition of iron to the *Dunaliella salina* culture medium stimulated *β*-carotene production. The iron enrichment in the *Chlorella vulgaris* culture could increase algal growth and lipid accumulation [24], where the total lipid content of algae grown in the medium supplemented with 1.2×10^−5^ M FeCl_3_ reached 56.6% of the dry biomass, which was a 3–7 fold increase compared to the medium without iron enrichment. Manganese is another important component in algal photosynthesis and also presents in enzymes to remove toxic superoxide radicals to sustain algal growth [25]. Chernikova *et al*. [26] reported that manganese (MnCl_2_) enhanced the capacity to accumulate inorganic minerals and catalysed protein synthesis in *Spirulina platensis*. Molybdenum is coupled with iron in the enzymes for nitrate reduction, and its deficiency diminishes the nitrate uptake mechanism and interferes with lipid synthesis [27]. Nickel can facilitate nitrogen uptake to enhance the growth of *Thalassiosira weissflogii* when urea is the nitrogen source, suggesting the positive role of Ni in enhancing algal growth [28]. Berges *et al*. [29] also reported that the addition of nickel and molybdenum to the algal culture medium increased the overall primary productivity. Coincidently, in a field survey, Wake and Hillen [3] found that wherever the *B. braunii* bloom occurred in the Darwin River reservoir, the nickel concentration in the environment was always higher than that in adjacent water bodies where no *B. braunii* bloomed, suggesting this trace element may trigger the occurrence of *B. braunii*. However, no laboratory testing has been conducted so far to test the need of nickel to enhance the growth of *B. braunii* in the laboratory since the early field survey work of Wake and Hillen’s in the 1980’s.

**Table 2:** Central composite design matrix and the responses of biomass and hydrocarbon production to Fe (*x_1_*), Mn (*x_2_*), Mo (*x_3_*) and Ni (*x_4_*).

Runs	Independent variables				Responses	
	Coded levels				Biomass(g/L)	Hydrocarbon (%, w/w)
	*x_1_*	*x_2_*	*x_3_*	*x_4_*		
1	1	1	1	1	0.246	14.82
2	−1	−1	1	1	0.292	14.31
3	1	−1	−1	1	0.251	15.45
4	−1	1	−1	1	0.296	14.56
5	1	−1	1	−1	0.124	13.99
6	−1	1	1	−1	0.120	13.42
7	1	1	−1	−1	0.136	14.83
8	−1	−1	−1	−1	0.125	13.86
9	1	−1	1	1	0.257	13.96
10	−1	1	1	1	0.320	14.12
11	1	1	−1	1	0.248	14.19
12	−1	−1	−1	1	0.306	14.00
13	1	1	1	−1	0.116	13.96
14	−1	−1	1	−1	0.121	15.26
15	1	−1	−1	−1	0.105	14.68
16	−1	1	−1	−1	0.126	13.96
17	1.72	0	0	0	0.215	20.23
18	−1.72	0	0	0	0.231	19.24
19	0	1.72	0	0	0.123	12.25
20	0	−1.72	0	0	0.121	11.59
21	0	0	1.72	0	0.118	18.57
22	0	0	−1.72	0	0.124	20.18
23	0	0	0	1.72	0.289	12.54
24	0	0	0	−1.72	0.094	11.90
25*	0	0	0	0	0.124	19.31
26*	0	0	0	0	0.120	18.46
27*	0	0	0	0	0.123	19.17
28*	0	0	0	0	0.127	20.13
29*	0	0	0	0	0.122	19.74
30*	0	0	0	0	0.126	18.45

* Central point values contributing to the degree of freedom for pure error calculation.

Optimization of micronutrient requirements is an important undertaking prior to the establishment of sustainable production of *B. braunii* on a large scale. The conventional method to optimise the level of multiple nutrients in algal culture has been focussed on one-factor-at-a-time approach, studying the effect of one nutrient on the response of algae by keeping the other nutrients constant. However, this approach is time consuming and does not take into account interactions between nutrients, which usually results in poor optimization results [30], [31].

**Table 3:** Analysis of variance (ANOVA) for the fitted quadratic polynomial regression model for optimization of the algal biomass production.

Source	Sum of squares	*df*	Mean square	*F*-value	Probability *P -*value
Model	0.162049	14	0.011575	31.64	<0.001
Residual	0.005488	15	0.000366		
Lack of fit	0.005354	10	0.000535	20.08	0.002
Pure error	0.000133	5	0.000027		
Cor. total	0.167537	29			
*R^2^* = 0.967					
Adj. *R^2^* = 0.937 Pred. *R^2^* = 0.824					

Techniques in experimental design are critical to identify key nutrients required for algal growth. In this study we used the response surface methodology (RSM) [32] to explore the requirement of micronutrients in the culture of *B. braunii* because the RSM approach can optimise the nutrient requirement with low input of time and resources [33], [34], [35]. This approach has been widely used in optimization of plant nutrients [36], [37], bacterial medium composition [38], enzymatic hydrolysis [39], [40], synthesis of polymers [41], food processing [42], [43] and operation conditions for photobioreactors [44]. The RSM approach has also been used for medium optimisation in algal culture. Azma *et al*. [45] optimised the culture medium for *Tetraselmis suecica* by RSM and increased algal production by two times. Similarly, by using RSM, Isleten-Hosoglu *et al*. [46] optimised the carbon and nitrogen concentrations for *Chlorella saccharophila* and improved biomass production by 7.7 fold.

The objectives of this study were to (1) estimate the roles of the four micronutrients iron, manganese, molybdenum, and nickel in regulating the responses of algal biomass and hydrocarbon, and (2) identify the optimum requirements of micronutrients for the cultivation of *B. braunii* to maximise hydrocarbon production.

**Table 4:** Analysis of variance (ANOVA) for the fitted quadratic polynomial regression model for optimization of the hydrocarbon production.

Source	Sum of squares	*df*	Mean square	*F*-value	Probability *P* value
Model	218.69	14	15.621	36.58	<0.001
Residual	6.406	15	0.427		
Lack of fit	4.127	10	0.413	0.91	0.584
Pure error	2.279	5	0.456		
Cor. total	225.096	29			
*R^2^* = 0.972					
Adj. *R^2^* = 0.945 Pred. *R^2^* = 0.875					

## Methods

### Materials and Procedures

*Botryococcus braunii* UTEX 572 was obtained from the University of Texas Culture Collection, USA. The basic macronutrients for algal growth were adapted from the Bold 3N medium, which also contains micronutrients including 5.35 µM Fe, 6.36 µM Mn, and 0.31 µM Mo [47]. All chemicals were of analytical regent grade. To avoid the effect of other unknown trace elements, soil residuals were not added into the medium in this study. The experiment for model construction was conducted at 24±1°C with illumination provided by fluorescent lights at 150 µmol/m^2^/s at 12 h light and 12 h dark. The algal growth experiments lasted 3 weeks.

**Table 5:** Concentration of micronutrients in different algal culture media.

Culture media	Micronutrients (µM)			
	Fe	Mn	Mo	Ni
Original Bold 3N	2.150	1.240	0.099	0.00
Modified Bold 3N-1	0.276	0.707	0.624	3.38
Modified Bold 3N-2	10.430	6.530	0.012	1.73

**Table 6:** Results of regression analysis of the full second-order polynomial model for optimization of algal biomass production with Fe (*x_1_*), Mn (*x_2_*), Mo (*x_3_*) and Ni (*x_4_*).

Model term	Coefficients estimated	*P*-value	*t*-Statistic
intercept	0.2196	<0.001	5.04
*x_1_*	−0.0433	<0.001	−5.93
*x_2_*	−0.0036	0.547	0.55
*x_3_*	−0.1471	0.249	−1.20
*x_4_*	−0.0058	0.795	−0.26
*x_1_x_2_*	−0.0001	0.999	−0.00
*x_1_x_3_*	0.0021	0.813	0.24
*x_1_x_4_*	−0.0043	0.019	−2.62
*x_2_x_3_*	0.0001	0.992	0.01
*x_2_x_4_*	−0.0003	0.798	−0.26
*x_3_x_4_*	0.0149	0.581	0.56
*x_1_^2^*	0.0044	<0.001	8.64
*x_2_^2^*	0.0004	0.290	1.10
*x_3_^2^*	0.1703	0.269	1.15
*x_4_^2^*	0.0294	<0.001	6.22

The dry weight of algal cells was measured by vacuum filtration onto pre-weighed Whatman® GF/C filters [48]. The filters with algal cells were freeze-dried, weighed, and expressed as algal biomass (g/L). Hydrocarbons in dry biomass were extracted on glass filters using *η*-hexane [48]. Solvents were removed from the extracts by a rotary evaporator and the residues were rinsed with *η*-hexane. Hydrocarbon fractions were purified by passing the samples through an alumina gel plug and eluting with *η*-hexane. Solvents were evaporated under a stream of nitrogen to dry, and the pure hydrocarbon fractions were measured gravimetrically and expressed as hydrocarbon content (%, w/w).

**Figure 2:**
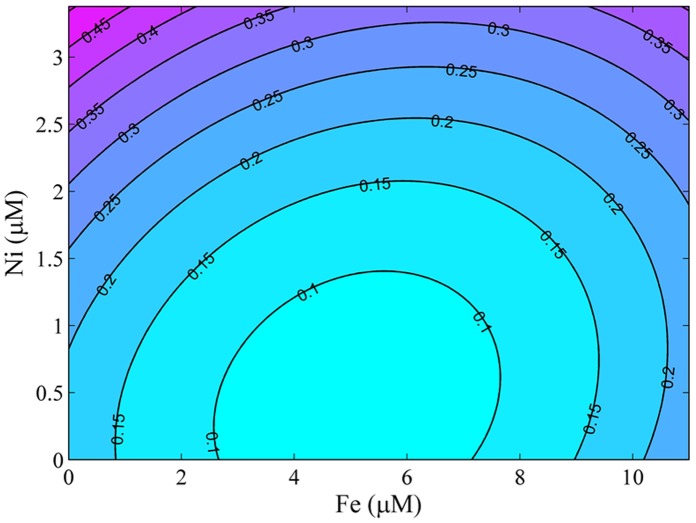
Contour plot showing biomass prediction from Fe (*x*_1_) Ni (*x*_4_) with other independent variables Mn (*x_2_*) and Mo (*x_3_*) being constant.

**Table 7:** Results of regression analysis of the full second-order polynomial regression model for optimization of hydrocarbon production with Fe (*x_1_*), Mn (*x_2_*), Mo (*x_3_*) and Ni (*x_4_*).

Model term	Coefficients estimated	*P*-value	*t*-Statistic
intercept	4.4600	<0.001	3.00
*x_1_*	−0.0082	0.974	−0.03
*x_2_*	2.3089	<0.001	11.46
*x_3_*	1.7040	0.690	0.41
*x_4_*	8.4303	<0.001	11.17
*x_1_x_2_*	0.0062	0.683	0.42
*x_1_x_3_*	−0.3608	0.245	−1.21
*x_1_x_4_*	0.0100	0.861	0.18
*x_2_x_3_*	−0.0720	0.768	−0.30
*x_2_x_4_*	0.0273	0.554	0.61
*x_3_x_4_*	−0.1031	0.911	−0.11
*x_1_^2^*	0.0120	0.497	0.70
*x_2_^2^*	−0.1865	<0.001	−16.22
*x_3_^2^*	−0.3860	0.940	−0.08
*x_4_^2^*	−2.5090	<0.001	−15.54

### Experimental Design

Central composite design (CCD) is one type of RSM approach [49] which allows estimating the polynomial regression between independent variables and dependant variables [50]. In this study, a 2^4^ CCD with 24 runs and six replications of the centre points were used to determine the optimal concentrations of iron, manganese, molybdenum, and nickel on the yield of algal biomass and hydrocarbon production (Fig. 1). The coded and corresponding actual values are given in Table 1. The corresponding central composite experimental design and their values are shown in Table 2. All the design points except the centre point (0, 0, 0, 0) were run in three replications. Due to the restriction of modeling protocol, only one mean value of the three replicates for each dependent variable was allowed to enter the model. Therefore, the degree of freedom of the triplicate for each non-centrepoint could not be used for pure error calculation. Experiments were repeated six times at the central point to provide an estimate of pure error [51], [52], [53], [54] thus providing adequate degree of freedom (*df* = 5) for pure error calculation (Tables 3 and 4).

**Figure 3:**
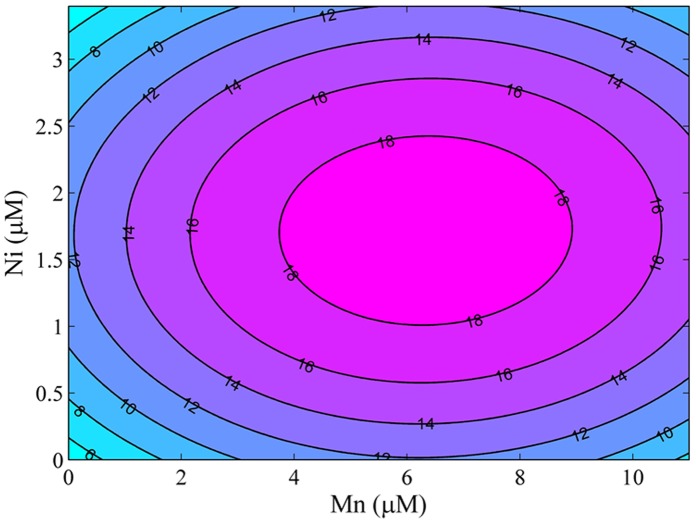
Contour plot showing hydrocarbon prediction from Mn (*x_2_*) and Ni (*x*_4_) with other independent variables Fe (*x_1_*) and Mo (*x_3_*) being constant.

**Figure 4:**
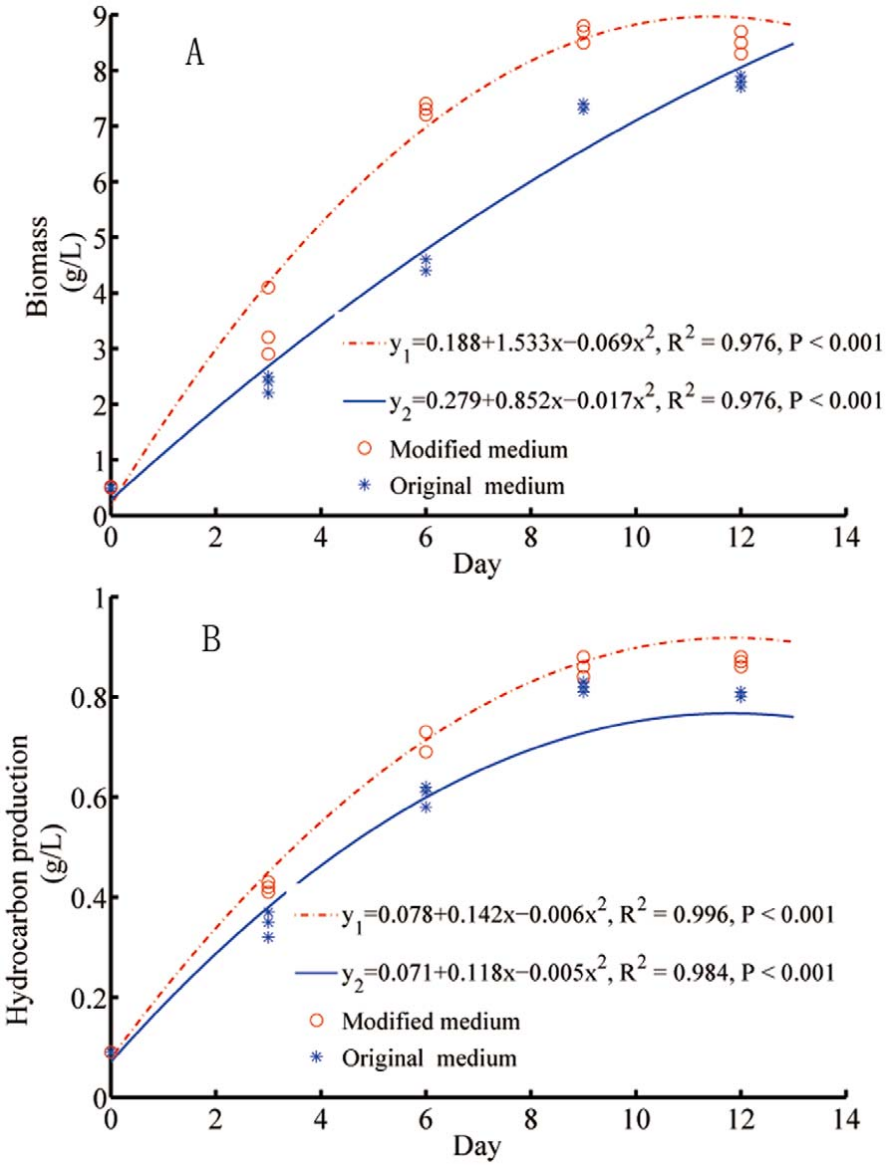
Regression plots of biomass (A) and hydrocarbon (B) productions in the modified and original Bold 3N media.

**Figure 5:**
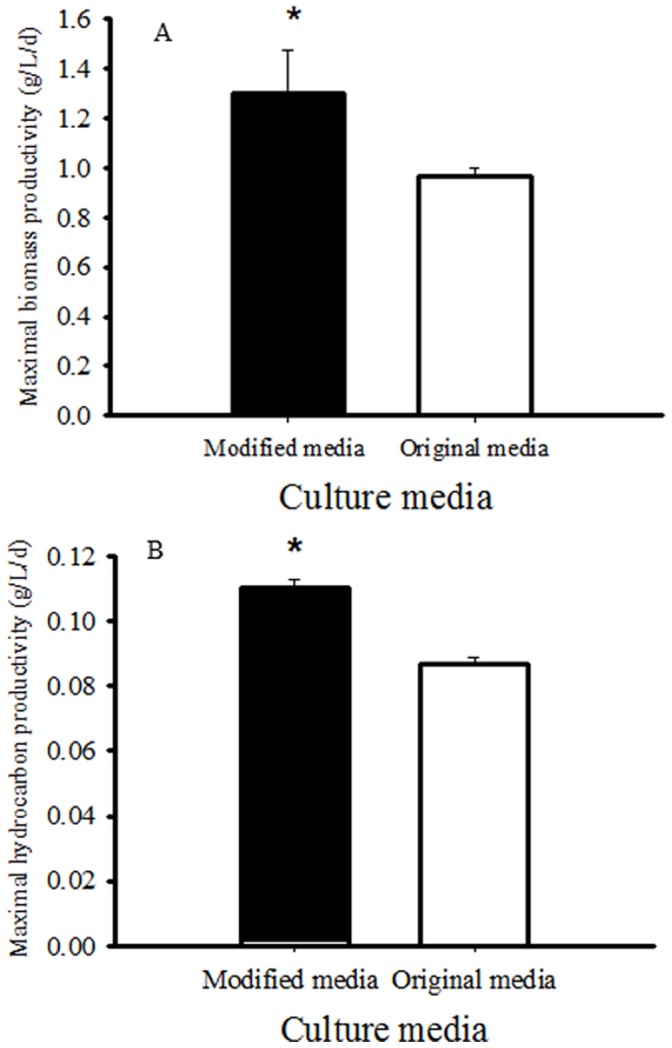
Comparison of maximal biomass (A) and hydrocarbon (B) productivities in the modified and original Bold-3N media.

Data from the CCD experiment were analysed by RSM. A mathematical model with a second-order polynomial regression was developed to describe the relationships between the predicted response variables (biomass or hydrocarbon) and the independent variables (Fe, Mn, Mo and Ni). The regression equation was described as follows (Eq. 1):
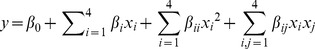
(1)where *y* is the predicted response variables (biomass or hydrocarbon production); *β*_0_ is a constant, *β*_i_ is the linear coefficient, *β*_ii_ is the quadratic coefficients, *β*_ij_ is the interaction coefficients of the model, respectively; *x_i_* and *x_j_* (*i = *1, 4; *j* = 1, 4; *i*≠*j*) represent the non-coded independent variables (micronutrient concentrations).

### Model Validation

The predicted models on algal biomass and hydrocarbon production of *B. braunii* were validated in an independent experiment using optimized micronutrient concentrations from the genetic algorithms calculations [55]. A flat plate photobioreactor (3.2 L) was used as the culture vessel under a light intensity of 300 µmol/m^2^/s and a mixing rate of 1.10 L/L/min. The *B. braunii* cells were separately inoculated into the original Bold 3N medium, the modified Bold 3N-1 medium for producing algal biomass, and the modified Bold 3N-2 for producing hydrocarbon with different micronutrient compositions (Table 5). The experimental protocols in the validation study were the same as those in the model construction. Algal biomass and hydrocarbon content were separately measured at 3-day intervals over 12 days to assess the response of algal performance to modified media. The productivities of algal biomass and hydrocarbon during the experimental period were also calculated and expressed as g/L/day. All data points in the figures were the mean of three replicates to provide a better estimate of the response of each dependent variable.

### Statistical Analysis

The data analyses for model construction were performed with MINITAB 16, based on the response surface methodology. The *F*-test for the analysis of variance (ANOVA) was performed on experimental data to evaluate the statistical significance of the model. The significance of regression coefficients was evaluated using *t*-test. The contour plots described by the regression model were drawn using MATLAB 7 to illustrate the effects of the independent variables and interactive effects of each independent variable on the response variables.

Optimisation of nutrient composition in the medium was determined by the procedure of genetic algorithms (MATLAB 7), which is a computer simulation program based on the best fit theory of natural selection to generate optimal solutions to problems [55]. In simulations, the program selected the best-fit concentration of each nutrient to maximise the algal response such as biomass and hydrocarbon production. In the validation experiment, data from the original 3N medium and modified medium were analysed by quadratic regression to compare the significant differences of curves. The probability level for significant difference was set at *P*<0.05.

## Results and Discussion

### Model Fitting

The application of RSM yielded the following regression equations for biomass (Eq. 2) and hydrocarbon production (Eq. 3). A central composite design (CCD) with five coded levels for all the four factors: iron, manganese, molybdenum, and nickel were used for model simulations. The range of variables, experimental designs and results for biomass and hydrocarbon production are presented in Table 2. The second order polynomial regression equations were used to fit the dependent variables (*Y*_biomass_ and *Y*_hydrocarbon_) to the independent variables *x*_1_ (iron), *x*_2_ (manganese), *x*_3_ (molybdenum) and *x*_4_ (nickel).
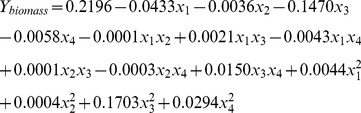
(2)

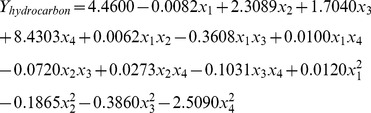
(3)


The significance and adequacy of the regression model were tested using ANOVA. These two regression models could significantly predict algal biomass (*P*<0.001) and hydrocarbon production (*P*<0.001) from the four micronutrients (Tables 3 and 4). The predicted *R^2^* (0.824 for Eq. 2 and 0.875 for Eq. 3) agreed well with the adjusted model *R^2^* (0.937 for Eq. 2 and 0.945 for Eq. 3), suggesting a close correlation between the observed values and the predicted values. Therefore, we can use the regression models to predict algal biomass and hydrocarbon production from the amount of micronutrients in the culture medium.

### Effect of Micronutrients on Algal Biomass

The regression coefficients of the model for biomass prediction are presented in Table 6. The linear effect of *x_1_* and the quadric effect of *x_1_^2^* and *x_4_^2^* had significant effects (*P*<0.001) on *Y*_bioamss_ followed by the interaction effect of *x_1_x_4_* (*P* = 0.019). Other terms of the model had no significant effect on *Y*_bioamss_. Negative coefficients of *x_1_* and interaction term *x_1_x_4_* decreased *Y*_bioamss_. However, the quadratic terms of *x_1_^2^* and *x_4_^2^* had positive effects on *Y*_bioamss_.

The interaction between two independent variables (Fe and Ni) and the response variable (biomass) was shown by the contour plots generated by keeping the independent variables (Mn and Mo) as constants (Fig. 2). The algal biomass was sensitive to the change of Fe and Ni concentrations. As the concentration of Ni increased, algal biomass increased progressively. The Fe in the medium at either low or high concentrations increased algal biomass when Ni concentrations were high.

In this study, the positive relationship between algal biomass and Ni concentrations corroborates an early report by Wake and Hillen [3] that the *B. braunii* bloom occurred in waters with the nickel concentration of 0.1 mg/L. In other studies, however, nickel accumulation in cells has been shown to cause a detrimental effect on algal growth as nickel is toxic to some physiological processes [56]. Wong *et al*. [57] reported that both *Chlorella vulgaris* and *Chlorella miniata* were capable of cell division after being treated with wastewater containing nickel for 24 h, but the growth rate was reduced in proportion to the concentrations of nickel in the wastewater. Despite this inhibition effect of nickel on other algal species, the present study does suggest that the use of nickel stimulated the growth of *B. braunii*.

### Effect of Micronutrients on Hydrocarbon Production

The regression coefficients of the model for hydrocarbon production are presented in Table 7. The linear effect of *x_2_* and *x_4_*, and the quadric effect of *x_2_^2^* and *x_4_^2^* had significant effects (*P*<0.001) on *Y*_hydrocarbon_. Other terms of the model had no significant effect on *Y*_hydrocarbon_. Positive coefficient of *x_2_* and *x_4_* indicated their role to enhance *Y*_hydrocarbon_. However, the quadratic terms of *x_1_^2^* and *x_4_^2^* had negative effects on *Y*_hydrocarbon_.

The interaction effects of two independent variables (Mn and Ni) on the response variable (hydrocarbon) are shown by the contour plots generated by keeping the independent variables (Fe and Mo) as constants (Fig. 3). Hydrocarbon production was more sensitive to the change of Mn and Ni concentrations. An increase in hydrocarbon production was observed with the increase of Mn concentrations. But this trend was reversed when the Mn concentration was above 9 µM. The effect of Ni on *Y*_hydrocarbon_ followed the similar trend. With the increase of Ni concentration, *Y*_hydrocarbon_ firstly increased and then decreased as a result of excessive Ni concentration. The circular profile of the contour plots indicated that the interaction between the Mn and Ni concentrations on hydrocarbon was negligible (Fig. 3).

The composition of the culture medium affects not only algal productivity, but also secondary metabolites [58]. This finding was consistent with result of Wang et al. [59] who found that the increase of Fe and Mn concentrations stimulated the growth of blue green algae, while a further increase in their concentrations inhibited algal growth. Cloëz *et al*. [60] found that lipid synthesis increased by three times after adding manganese, copper and nickel at 2 mM. On the other hand, Mohammady and Fathy [61] reported that the total lipid content in *Dunaliella salina* cultivated in nickel supplemented media (0.5 mg/L NiCl_2_) has reduced in comparison to the control. In another study, Rousch and Sommerfeld [62] found that manganese had stronger impact on the growth of a green alga (*Ulothrix* sp.) than nickel. However, in this study, both nickel and manganese regulated the production of hydrocarbon, though the algal biomass was only affected by nickel.

### Optimisation of Micronutrients

The concentrations of these four micronutrients for producing algal biomass were optimized using the genetic algorithm calculation. The optimal medium for biomass consisted of 0.266 µM Fe, 0.707 µM Mn, 0.624 µM Mo and 3.38 µM Ni. By running the optimization simulation within the experimental range, the optimal medium for hydrocarbon production is recommended to contain 10.43 µM Fe, 6.53 µM Mn, 0.012 µM Mo and 1.73 µM Ni. It is worth noting that the optimal composition of these four micronutrients for algal biomass was different from that for hydrocarbon production. This difference highlights the importance of selecting culture medium to achieve different objectives in algal culture since the nutrient requirement differs for algae cell division and accumulation of secondary metabolites [63].

### Validation of Algal Growth and Hydrocarbon Production

The reliability of nutrient requirement generated from the predicted models and the genetic algorithm calculations for biomass and hydrocarbon production in *B. braunii* were validated in an independent photobioreactor study. From day 3 to day 12, the algal biomass produced in the Bold 3N medium supplemented with 0.266 µM Fe, 0.707 µM Mn, 0.624 µM Mo, 3.38 µM Ni was significantly higher than that produced in the original Bold 3N medium (*P*<0.05, Fig. 4A). The maximal algal biomass productivity (1.300±0.176 g/L/day) in dry weight with modified media was significantly higher than that (0.967±0.033 g/L/day) in the original media (*P*<0.05, Fig. 5A).

The hydrocarbon production of algae in the Bold 3N medium supplemented with 10.43 µM Fe, 6.53 µM Mn, 0.012 µM Mo and 1.73 µM Ni was significantly higher than that in the original medium from day 3 to day 12 (*P*<0.05, Fig. 4B). The maximal hydrocarbon productivity (0.110±0.003 g/L/day) in the modified media was significantly higher than that (0.087±0.002 g/L/day) in the original media (*P*<0.05, Fig. 5B).

The biomass and hydrocarbon productivity are key parameters affecting the economic feasibility of producing bioproducts from algae. The micronutrient concentrations optimised by modelling were validated in a photobioreactor, and the accuracy and reliability of the model in predicting nutrient requirements for producing algal biomass and hydrocarbon have been confirmed.

### Conclusion

The application of response surface methodology (RSM) is a reliable approach to model and optimize the requirements for iron, manganese, molybdenum, and nickel in producing algal biomass and hydrocarbon in *B. braunii.* Nickel and iron played significant roles but manganese and molybdenum had a trivial role in algal biomass production. In contrast, nickel and manganese were more important than molybdenum and iron in regulating algal hydrocarbon production. The production of algal biomass and production of hydrocarbon require different micronutrients in the culture medium. The recommended levels of micronutrients in the Bold 3N medium are 0.266 µM iron, 0.707 µM manganese, 0.624 µM molybdenum and 3.38 µM nickel for *B. braunii* biomass and 10.43 µM iron, 6.53 µM manganese, 0.012 µM and 1.73 µM nickel for hydrocarbon production. The model validation showed that by using modified algal culture media, algal biomass productivity increased 1.345 fold and hydrocarbon productivity increased 1.274 fold compared with the original Bold 3N medium without addition of the trace elements.
